# The Construct Validity of the ICD-11 Severity of Personality Dysfunction Under Scrutiny of Object-Relations Theory

**DOI:** 10.3389/fpsyt.2021.648427

**Published:** 2021-07-22

**Authors:** Amin Nazari, Steven K. Huprich, Azad Hemmati, Farzin Rezaei

**Affiliations:** ^1^Department of Psychology, University of Kurdistan, Sanandaj, Iran; ^2^Department of Psychology, University of Detroit Mercy, Detroit, MI, United States; ^3^Department of Psychiatry and Behavioral Sciences, College of Human Medicine, Michigan State University, Lansing, MI, United States; ^4^Neurosciences Research Center, Research Institute for Health Development, Kurdistan University of Medical Sciences, Sanandaj, Iran

**Keywords:** ICD-11, severity of personality dysfunction, object-relations theory, STIPO-R, LPFS-SR

## Abstract

The current classification of personality disorder in ICD-11 includes a description of personality functioning, derived from a number of theoretical paradigms, but most notably consistent with the psychodynamic approach. Concurrently, an object-relations model of personality functioning in a dimensional assessment of severity is provided in the Structured Interview of Personality Organization-Revised (STIPO-R). To date, there are no published measures of International Classification of Diseases-11 (ICD-11) personality severity, though the construct is very comparable to the concepts assessed in the Diagnostic and Statistical Manual of Mental Disorders-5 (DSM-5) levels of personality functioning concept, which is measured by the Level of Personality Functioning Scale-Self-Report (LPFS-SR). This study examined the validity of ICD-11 personality functioning, as measured by the LPFS-SR, by evaluating its associations with the STIPO-R in Kurdistan region. The samples included 231 University students and 419 inpatient participants across four hospitals (267 with a diagnosed personality disorder). All the components of LPFS-SR and STIPO-R were positively and significantly intercorrelated. The components of each measure discriminated PD and non-PD patients from a University, non-clinical group adequately. Despite slightly better performance of the STIPO-R in this discrimination, the measures had a high congruence in predicting personality dysfunction. Overall, the findings of the present study support the validity of ICD-11 construct for evaluating personality functioning.

## Introduction

Personality disorder includes impairments in functioning of aspects of the self (i.e., identity, accuracy of self-view, self-worth, self-direction), and problems in interpersonal functioning (e.g., parent–child, romantic relationships, school/work, family, friendships, peer contexts) (1). The level of severity of personality disorder was recently incorporated into International Classification of Diseases (ICD-11) as a means of classifying personality functioning and assigning patients a personality disorder diagnosis (2). The level of personality functioning has been derived from multiple frameworks (2, 3), including psychodynamic, interpersonal, and personological. These three paradigms have consistently stressed the dynamics of the intrapersonal and interpersonal (3).

The International Classification of Diseases-10 (ICD-10) and the Diagnostic and Statistical Manual of Mental Disorders-IV (DSM-IV) had no account of severity of dysfunction (4). By contrast, The ICD-11 model of personality disorder puts the severity level at the first line of personality disorder diagnosis, which has been theoretically and empirically central in understanding a patient's overall level of functioning (5–9). The degree of severity of personality pathology, in both diagnostic systems of DSM-5 AMPD and the ICD-11 is defined similarly and can be evaluated by measures like LPFS-SR (1, 10). By including severity in the conceptualization and diagnosis of personality disorder, studies have found that researchers and clinicians have more adequate prediction and prognosis in the assessment of personality disorder (5, 6, 9, 11).

The ICD-11 levels of severity of personality disorder are congruent with personality organization in the psychodynamic approaches (1, 12–18). Waugh et al. (19) stated that the concept of “psycho-structural level” contained within the psychodynamic model (20) shows many parallels with the Level of Personality Functioning Scale-Self-Report (LPFS) as described in Criterion A of DSM-5 Alternative Model of Personality Disorder (AMPD) assessment, which refers to levels of personality functioning as assessed across the domains of self and interpersonal relatedness. Ferrer et al. (21) also claimed that LPFS dimensions could be traced back to Kernberg model of personality organization (PO) (14, 22). Even prior to the publication of the DSM-5 and ICD-11, Huprich (23) argued object relations theory and psychodynamic models ought to be considered for future models of personality pathology, given their appealing clinical utility, integration with other models of psychological functioning, and empirical support in the literature.

Object relations theory (21) is probably the first theoretical approach in psychology that considered a dimensional view toward personality in its classification of personality disturbances from psychosis to borderline and neurotic. According to this framework, neurotic personality organization (produced from repression-based defense mechanisms) is the highest level of functioning and approximates what might be considered “normal personality.” Borderline personality organization (BPO), which mainly includes most personality disorder features, describes mental conditions of immature people with a lower level of integrated and complex representations of self and other. BPO is presented in Kernberg's model (13, 21), through two dimensional continuums of severity and introversion-extroversion. Unlike psychotic levels of organization, and as the greatest difference between the two classes of mental disturbance, patients with BPO do not lose their reality testing. They organize the relational patterns, and their inflexibility and immaturity lead to self and interpersonal dysfunctioning.

The Structured Interview of Personality Organization (STIPO) (24) was developed from object relations theory as a dimensional assessment tool to assess severity of personality pathology. The STIPO organizes three levels for personality organization (PO) within a continuum of severity, from psychotic to borderline (with high and low levels) and neurotic, and from internalizing to externalizing (14, 18). Clarkin et al. (25) provided a revised version (STIPO-R) to achieve a structural diagnosis by thoroughly evaluating the essential concepts of identity, object-relations (ORs), defenses (primitive and higher-level), aggression, moral values and narcissism (18, 25). Among these critical concepts, identity (as the main definition of self) and aggression (as the principal part of interpersonal conflicts) have the main role in self and interpersonal aspects of personality; especially in personality disorder. Through an objective and standard method of collecting information about the severity of personality pathology, the STIPO-R also provides valuable data about the probability of dropping out of treatment (26), comorbidities (27, 28), the prognosis of treatment, and an adjustment in treatment planning based upon the level of personality organization (29). The STIPO has also been evaluated for its reliability and validity, which has received solid empirical support (13, 30, 31).

Recent studies (20, 22, 30) have explored the congruence of the levels of personality functioning construct (i.e., criterion A of AMPD) with the STIPO, which is very similar to the ICD-11 model of personality functioning. Especially, Ferrer et al. (21) and Hörz-Sagstetter et al. (29) suggested STIPO was a valid instrument for the evaluation of personality functioning for further studies. Kampe et al. (32) also demonstrated a close correspondence between the approach to assessing personality pathology adopted in the LPFS (operationalized by the SCID-AMPD) and that used in the psychodynamic concept of personality organization (operationalized by the STIPO).

Given that there have been demonstrated empirical relationships between the LPFS and the STIPO-R, the present study was conducted to confirm what has been published in past studies, but also to lay out the groundwork for the value and utility of the levels of personality functioning framework that has been articulated in ICD-11. The present study extends the literature by evaluating these relationships in large Iranian samples of patients and University students. Since past studies have found strong correspondence of the personality functioning concept with the STIPO-R, the present study would extend the empirical support for the psychodynamic underpinnings of this concept.

## Method

### Participants

This study included both University and clinical samples. The University sample included 347 students attending University of Kurdistan, who voluntarily consented to participate. The inclusion criteria for this sample were voluntary participation and age ≥18. The exclusion criteria were having a diagnosed mental disorder and/or abusing substances or medications, for which 64 students were excluded. The clinical participants involved 739 inpatients, who were previously assigned psychiatric disorder diagnoses, except for psychotic disorders, by experienced psychiatrists through structured diagnostic interviews based on DSM-5 criteria. These patients were hospitalized at four psychiatric hospitals. The inclusion criteria for the clinical sample were voluntary participation, age ≥18, and a diagnosis of a psychiatric disorder except for psychotic disorders. The researchers did not have access to information about potential comorbidities (particularly substance and/or alcohol use disorders).

**Figure 1:**
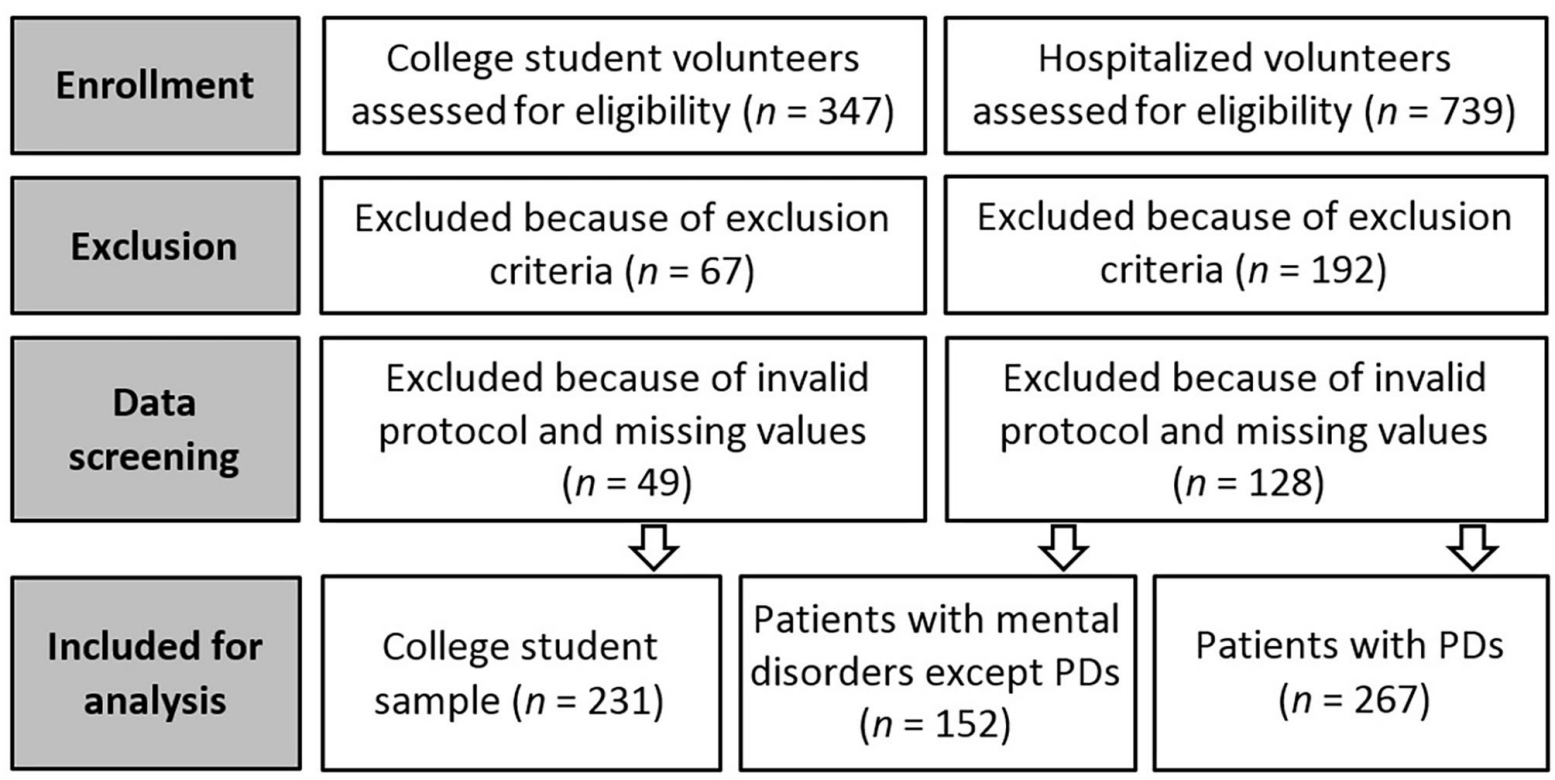
Flow diagram for processes of the enrollment, exclusion and inclusion of samples for analysis.

Per preliminary data screenings, protocols which were invalid (e.g., similar responses to all items, not responding to one of the measures) and/or included more than 10% non-response items were eliminated to avoid biased statistical analyses (33). A total of 650 valid final protocols were identified. Among the University sample, 231 protocols were determined to be valid for the final analysis phase, of which 138 (59.7%) were female and 92 (39.8%) were male. The final valid clinical sample included 419 hospitalized participants, who assigned into groups: the patients with personality disorders (PDs; *n* = 267) and patients with psychiatric disorders except PDs (*n* = 152). The predominant PD diagnosis among the clinical group was Borderline PD (198 patients). Figure 1 presents the flow diagram for processes of the enrollment, and exclusion/inclusion of participants for analysis. Supplementary Table 1 presents the demographic data for all the participants and elaborates the detail information about clinical samples. The Research Ethics Committee of the University of Kurdistan approved this study (IR.UOK.REC.1397.014), and all respondents provided written informed consent prior to data collection.

### Measures

#### Structured Interview of Personality Organization-Revised (STIPO-R) (25)—Persian Version

The STIPO-R is a semi-structured interview constructed to evaluate the structural domains of personality functioning that are central to understanding the individual from an object relations model of personality and personality pathology based on Kernberg's psychodynamic personality organization concept (22, 34). The STIPO-R contains 55 items covering five domains of functioning: identity, object relations, defenses, aggression, and moral values, for measuring personality organization through seven dimensions of personality: identity integration, quality of object relations, use of primitive defenses, quality and nature of aggression, adaptive coping versus character rigidity, moral values, and reality testing (18, 25). The STIPO-R also has scoring for a narcissism dimension. This interview provides the clinician and researcher with dimensional scores on key domains of personality functioning. The severity of dysfunction in each domain can be used by the clinician for treatment planning and by the researcher for selection of subjects and measurement of change in relation to treatment interventions. The standardized format and scoring system allow the interviewer to rate the subject's responses (0 = absent; 1 = subthreshold; or 2 = present) at the individual item level as the interview proceeds. Once the interview is completed, the scores at the individual item level are summed within each domain to give a total domain score. For the rating procedure, satisfactory inter-rater reliability has been found (30, 31). The STIPO has demonstrated satisfactory psychometric properties for the domains of object relations theory in different clinical samples (13, 30, 35, 36), which can be reliably used in both genders and culturally diverse contexts (12, 29, 36). Preti et al. (26) also reported a significant association between STIPO structural characteristics and DSM diagnoses. In this study, Cronbach's alphas for the seven domains were 0.80 (identity), 0.87 (object-relations), 0.72 (primitive defenses), 0.41 (high-level defenses), 0.85 (aggression), 0.86 (moral values), 0.70 (narcissism), and 0.90 (total score).

##### Translation Procedure

The translation from English to Persian was performed according to advised procedures (37, 38). Equivalence with the original intended meaning of the items was the guiding principle in the translation process. First, the STIPO-R items were independently translated into Persian by a four-member team, including an English language specialist, a psychiatrist (the fourth author), a psychologist, and a psychometrics specialist (the third author). The preliminary Persian version of the STIPO-R was given to a professional translator, blinded to the original English version, for back-translation to English. The English back-translation was then sent to the owners of the STIPO-R (25) for review. Finally, 11 items deemed to be semantically different from the English original were modified under supervision of the authors of the STIPO-R. The final confirmed Persian translation of the STIPO-R was presented as a pilot implementation to five interviewers, and the items were determined to be clear and comprehensible.

### Level of Personality Functioning Scale-Self-Report (LPFS-SR)-Persian Version

The LPFS-SR (39) is an 80-item self-report instrument that assesses disturbances in self and interpersonal functioning on a global severity continuum, representing Criterion A of the AMPD of DSM-5 Section Results. The LPFS-SR comprises four personality function components, including Identity (21 items) and Self-Direction (16 items) as subsets of self-functioning, and Empathy (23 items) and Intimacy (20 items) as subsets of interpersonal functioning. The measure utilizes a four-point response scale (1 = *Totally False, not at all True*; 2 = *Slightly True*; 3 = *Mainly True*; and *4* = *Very True*). The current study used the algorithm provided by Morey (39) for weighting item scores [weights ranging from −0.5 for Level 0 [“*little or no impairment”*] items to +3.5 for Level 4 [“*extreme impairment”*] items]. Hemmati et al. (40), in a validation study of Persian translation of LPFS-SR, confirmed its high internal consistency and found that it significantly discriminated between the non-clinical and clinical samples. Additionally, Morey et al. (39) found support for a single factor structure of personality dysfunction. In the current study, Cronbach's alphas for the four domains were 0.86 (Identity), 0.86 (Self-Direction), 0.81 (Empathy), 0.85 (Intimacy), and 0.96 (total score).

### Statistical Analyses

Zero-order correlations were calculated for evaluating the association between each of the LPFS-SR and STIPO-R components (Table 1). Independent samples *t*-tests and one-way analysis of variance (ANOVA) along with the Cohen's *d* (41) were applied for comparing the means of LPFS-SR and STIPO-R components among the groups (Table 2). Binomial (Table 3) and Multinomial (Table 4) logistic regression analyses were conducted with LPFS-SR and STIPO-R components as the independent variables, and group membership (PD, Borderline PD, non-PD patients, and non-clinical subjects) as the dependent variables. All statistical analyses were performed through the IBM-SPSS©-24 software.

**Table 1:** Bivariate correlations of LPFS-SR and STIPO-R components (*n* = 650).

**Measures**	**1**	**2**	**3**	**4**	**5**	**6**	**7**	**8**	**9**	**10**
**STIPO-R**										
1. Identity										
2. Object-relations	0.67									
3. Lower-level-defenses	0.67	0.60								
4. Higher-level-defenses	0.49	0.47	0.58							
5. Aggression	0.71	0.62	0.70	0.51						
6. Moral values	0.55	0.58	0.56	0.42	0.75					
7. Narcissism	0.67	0.67	0.71	0.52	0.57	0.52				
**LPFS-SR**										
8. Identity	0.62	0.47	0.64	0.46	0.60	0.47	0.53			
9. Self-direction	0.64	0.47	0.61	0.44	0.61	0.47	0.49	0.86		
10. Empathy	0.61	0.48	0.58	0.42	0.58	0.47	0.48	0.83	0.84	
11. Intimacy	0.62	0.50	0.61	0.40	0.59	0.46	0.51	0.86	0.84	0.88

*All values are significant at p < 0.001*.

**Table 2:** Independent Samples *T*-tests and one-way ANOVAs comparing means of the LPFS-SR, STIPO-R components between groups.

**Measures**	**PD vs. non-clinical**			**BPD vs. non BPD patients**			**PD, Non-PD and non-clinical**	
	**Mean difference**	***t***	**Cohen's *d***	**Mean difference**	***t***	**Cohen's *d***	**F**	**Partial Eta squared**
***STIPO-R***								
Identity	10.06	25.30	2.24	3.96	8.73	0.85	328.95	0.50
Object-Relations	6.23	17.78	1.73	1.83	4.18	0.41	152.33	0.32
Lower-Level-Defenses	3.81	16.86	1.50	1.75	6.44	0.63	139.88	0.30
Higher-Level-Defenses	2.03	12.72	1.15	0.60	3.14**	0.31	77.93	0.19
Aggression	6.90	22.71	1.99	4.20	10.72	1.05	239.99	0.43
Moral Values	3.49	14.18	1.24	1.61	4.97	0.49	99.43	0.24
Narcissism	4.62	14.53	1.29	2.01	5.52	0.54	110.69	0.25
***LPFS-SR***								
Identity	31.81	14.66	1.37	12.72	5.35	0.56	122.09	0.30
Self-Direction	29.14	15.97	1.49	9.85	4.64	0.48	143.30	0.33
Empathy	20.86	16.09	1.50	4.27	2.81**	0.30	142.68	0.33
Intimacy	30.68	16.14	1.50	7.06	3.17**	0.33	145.93	0.33

*All values are significant at p < 0.001, but three flagged values that are significant at p < 0.01. PD patients (n = 267), Non-PD patients (n = 152) and non-clinical (n = 231)*.

**Table 3:** Binomial logistic regression analysis of group membership predicted by LPFS-SR and STIPO-R components.

	**Model**	**Predictor**	***B (S.E.)***	***Wald***	***Exp. B (C.I.)***	**Sensitivity**	**Specificity**	**Overall model significance**			***Phi* (Φ)**
								*****χ**^2^* (d.f.)**	**Nagelkerke *R^**2**^***	**Cox & Snell *R^**2**^***	
PD vs. Non-clinical	Model 1; STIPO-R	Identity	−0.35 (0.05)	46.70^***^	0.70 (0.63–0.78)	87%	90 %	427.53^***^ (7)	0.77	0.58	0.60^***^
		Object-relations	−0.15 (0.06)	6.75^**^	0.86 (0.77–0.97)						
		Lower-level-defenses	0.05 (0.09)	0.32	1.05 (0.88–1.27)						
		Higher-level-defenses	−0.04 (0.10)	0.16	0.96 (0.79–1.17)						
		Aggression	−0.34 (0.07)	20.91^***^	0.71 (0.62–0.83)						
		Moral values	0.11 (0.09)	1.52	1.11 (0.94–1.32)						
		Narcissism	0.08 (0.07)	1.11	1.08 (0.94–1.25)						
	Model 2; LPFS-SR	Identity	0.00 (0.01)	0.05	1.00 (0.98–1.02)	78 %	79 %	215.83^***^ (4)	0.50	0.38	
		Self-direction	−0.03 (0.01)	6.05*	0.97 (0.95–0.99)						
		Empathy	−0.03 (0.02)	4.66^*^	0.97 (0.94–1.00)						
		Intimacy	−0.03 (0.01)	5.92^*^	0.97 (0.95–0.99)						

*Exp. B, Exponential B (These are the odds ratios for the predictors)*;

* *p < 0.05*.

** *p < 0.01*.

*** *p < 0.001*.

*Phi (Φ) correlation of the models' group membership*.

**Table 4:** Multinomial logistic regression analysis of group membership predicted by LPFS-SR and STIPO-R components.

**Model**	**Predictor**	**Likelihood ratio tests**						**Overall model significance**		
		**PD vs. Non-clinical**			**Axis I vs. Non-clinical**			*****Φ**^2^* (d.f.)**	**Nagelkerke *R^**2**^***	**Cox & Snell *R^**2**^***
		***B (S.E.)***	***Wald*** **(d.f**. **=** **1)**	***Exp. B (C.I.)***	***B (S.E.)***	***Wald*** **(d.f**. **=** **1)**	***Exp. B (C.I.)***			
Model 1; STIPO-R	Identity	0.40 (0.05)	68.57^***^	1.49 (1.36–1.64)	0.37 (0.05)	62.61^***^	1.44 (1.32–1.58)	540.65^***^ (14)	0.64	0.57
	Object-relations	0.14 (0.05)	7.12^**^	1.15 (1.04–1.27)	0.13 (0.05)	6.65^**^	1.14 (1.03–1.26)			
	Lower defenses	0.03 (0.09)	0.08	1.03 (0.87–1.21)	0.01 (0.08)	0.03	1.01 (0.86–1.19)			
	Higher defenses	0.02 (0.09)	0.07	1.02 (0.86–1.21)	0.14 (0.08)	3.02	1.15 (0.98–1.35)			
	Aggression	0.35 (0.07)	25.84^***^	1.42 (1.24–1.62)	0.17 (0.07)	6.11*	1.18 (1.04–1.35)			
	Moral values	−0.12 (0.08)	2.24	0.88 (0.75–1.04)	−0.15 (0.08)	3.25	0.86 (0.74–1.01)			
	Narcissism	−0.06 (0.07)	0.71	0.94 (0.83–1.08)	−0.11 (0.07)	2.70	0.90 (0.79–1.02)			
Model 2; LPFS-SR	Identity	0.00 (0.01)	0.08	1.00 (0.98–1.02)	−0.01 (0.01)	0.80	0.99 (0.97–1.01)	254.94^***^ (8)	0.40	0.35
	Self-direction	0.03 (0.01)	7.34^**^	1.03 (1.01–1.05)	0.04 (0.01)	9.19^**^	1.04 (1.01–1.06)			
	Empathy	0.03 (0.02)	3.30	1.03 (1.00–1.06)	0.03 (0.02)	2.25	1.03 (0.99–1.06)			
	Intimacy	0.03 (0.01)	6.57^*^	1.03 (1.01–1.05)	0.03 (0.01)	4.44^*^	1.03 (1.00–1.05)			
Phi (Φ) correlation of the models' group membership	0.51^***^									

*Exp B, Exponential B (These are the odds ratios for the predictors)*;

* *p < 0.05*.

** *p < 0.01*.

*** *p < 0.001*.

## Results

As Table 1 shows, all dimensions of LPFS-SR and STIPO-R components were meaningfully correlated. The inter-component correlations for STIPO-R varies from 0.42 to 0.71, while the inter-component correlates for the LPFS-SR were 0.83 to 0.88. These high inter-component correlations for the LPFS-SR dimensions are indicative of the unitary nature of the LPFS-SR (42). Among seven components of STIPO-R, identity (ranging from 0.61 to 0.64), primitive defenses (ranging from 0.58 to 0.64), and aggression (ranging from 0.58 to 0.61) had the highest associations with the LPFS-SR components. Otherwise, all the LPFS-SR components had similar patterns of correlation coefficients with the STIPO-R components (values within the mid −0.40 range).

Overall, as Table 2 to illustrates, the discriminative capacities of the STIPO-R components (Cohen's *d* from 1.15 to 2.24) and the LPFS-SR dimensions (Cohen's *d* from 1.37 to 1.50) are considerable for differentiating PD patients from non-clinical participants. Mean differences are prominently high in Identity (*d* = 2.24) and Aggression (*d* = 1.99) of STIPO-R. The effect sizes for the other components are not as strong, though still in the medium to large range, for both STIPO-R (Cohen's *d* from.31 to 1.05) and the LPFS-SR (Cohen's *d* from.33 to.56) in discriminating BPD and non-BPD patients (all other patients with and without a PD). However, Aggression (*d* = 1.05) has still a sizeable effect in differentiating these groups. Furthermore, the results for comparing the means of three groups of PD, non-PD patients, and non-clinical subjects indicate the relatively lower effect sizes for the STIPO-R (partial eta-squared values ranging between 0.19 and 0.50) and the LPFS-SR (partial eta-squared values ranging between 0.30 and 0.33). Notably, Identity and Aggression had large effect sizes.

Two binomial logistic regression models were performed to compare the relative effects of the STIPO-R and LPFS-SR components on predicting the likelihood of group membership (PD vs. non-clinical). Model 1 contained the STIPO-R components as the predictor variables, which was significant, χ*2* (7, *N* = 398) = 427.53, *p* < 0.001, indicating the model's ability in distinguishing PD from non-clinical groups. This model explained between 58% (Cox and Snell *R*^2^) and 77% (Nagelkerke *R*^2^) of the variance in predicting PD presence and had very solid sensitivity (0.87) and specificity (0.90). Model 2 with LPFS-SR components as the predictor variables was also significant, χ*2* (4, *N* = 398) = 215.83, *p* < 0.001, indicating the model's ability in distinguishing PD from non-clinical groups. This model, with solid sensitivity (0.78) and specificity (0.79), explained between 38% (Cox and Snell *R*^2^) and 50% (Nagelkerke *R*^2^) of the variance in predicting PD likelihood. The Phi coefficient (ϕ = 0.60) indicated a high contingency between predicted group membership by the models.

The relative effects of the STIPO-R and LPFS-SR components were compared through multinomial logistic regression models for distinguishing PD and non-PD patients from non-clinical groups. According to Table 4, model 1 contained the STIPO-R components as the predictor variables, and significantly [χ*2* (14, *n* = 650) = 540.65, *p* < 0.001] predicted the likelihood of group membership (PD, non-PD patients, and non-clinical subjects) with 57% (Cox and Snell *R*^2^) and 64% (Nagelkerke *R*^2^) ability to explain the variances. Model 2 evaluate the LPFS-SR's ability to differentiate the same groups. Results indicate that the model was able to explain 35% (Cox and Snell R^2^) and 40% (Nagelkerke *R*^2^) of the variances, which was statistically significant [χ*2* (8, *n* = 650) = 254.94, *p* < 0.001]. Specifically, the model distinguished PD and non-PD patients from non-clinical groups. The Phi coefficient (ϕ = 0.51) indicated a high contingency between predicted group membership by the models.

## Discussion

Object-relations psychodynamic models of personality have posited that those with a personality structure that is composed of a strong sense of self (by way of self-esteem, identity, purpose, and overall agency) and one's ability to relate optimally to others (by way of being understanding and empathic, developing cooperation, respect, and closeness) is central to adaptive human functioning and an overall good quality of life. It also is the case that the affective and motivational bonds between others must preserve one's own needs as well as the needs of others, even when such needs come in conflict. When self, other, and affect (broadly defined) are performing in non-integrated, non-mutually informative ways, the individual is likely to have difficulties, thus leading to poor functioning. Such ideas are embedded in Kernberg's model of personality (43) and ideas of personality organization, and were central in the development of the STIPO-R.

It is thus gratifying to those who practice from the psychodynamic perspective to see the AMPD and ICD-11 incorporate central ideas of object relations theory and personality organization into the assessment and diagnosis of personality pathology. More so, it is especially helpful to recognize that when self, other, and affective/motivational aspects of this dyad operate in suboptimal ways, the more pathological a person will appear and the poorer their functioning will be. The present study lends support to these ideas.

Overall, we found that those with poor levels of personality functioning, as assessed by various components of the STIPO-R and LPFS, tend to have personality disorder diagnosis. Even without a personality disorder diagnosis, poor levels of personality functioning were associated with psychopathology in a wide range of patients, thus demonstrating the centrality of object relations and personality organization as a core component of overall personality functioning.

When comparing group mean differences, the largest effect sizes were seen for the STIPO-R Identity and Aggression components. However, all STIPO-R and LPFS-SR dimensions were able to successfully differentiate the PD patients, non-PD patients, and University student controls. Looking more specifically at pairwise group differences, it appears all STIPO-R and LPFS-SR dimensions yielded large effect sizes, clearly demonstrating the utility of both measures in differentiating patients of various levels of psychopathology. However, the strongest effects in these comparisons, as well as in the comparison of Borderline PD and non-Borderline PD patients, were observed in the STIPO-R Identity and Aggression dimensions. These dimensions can be seen as central to an object relations model of personality, as delineated by Kernberg (43). Specifically, Identity is that part of the self-representation that illuminates individuals' ideas about who they are, and Aggression is an emotional and behavioral manifestation of the quality of interpersonal relationships. When there are problems in the clarity of one's identity, coupled with problems in the expression of aggression, a person is likely to have difficulties in personality functioning, namely by way of severity of problems.

Interestingly, the decrease in magnitude of the effect sizes seemed to change consistently when moving from a University control group to a non-PD patient control group (as compared to the PD group). These findings demonstrate that that uniform changes across levels of psychopathology are associated with changes in personality functioning and, more broadly, personality organization. Thus, personality organization that is more integrated, structured, and nuanced has less severity and psychopathology. However, it would be noted that the largest magnitude of differentiation across groups occurred with the STIPO-R when compared with the LPFS-SR. There are likely two reasons for this. First, the STIPO-R assesses a broader range of functioning than the LPFS-SR. While the latter focuses exclusively upon self and other representations, the STIPO-R assesses defenses, the implementation of moral values, and narcissism, in addition to identity and object relations (which are components of the LPFS-SR). Second, the STIPO-R is an interview-based measure, which relies on the clinical expertise of trained interviewers, whereas the LPFS-SR is a self-report instrument that participants complete. Given the limitations of self-report measures (44), an interview-based measure is more likely to detect problems in psychological functioning that may not be assessed in an individual's self-report (45).

The current study also tried to extend the findings of the relation between the STIPO-R and the personality functioning operationalized by ICD-11 model of PD in a culturally different and large clinical and non-clinical sample. Given the use of the STIPO-R and LPFS-SR primarily in North American and European samples, the replication of past findings in this sample demonstrates the generalizability of previous findings and the utility of studying personality functioning in diverse, worldwide samples. Ongoing international efforts are needed in order to maximize the clinical utility of the ICD-11.

## Limitations

The most significant limitation to the present study is that the LPFS-SR was used as an approximation of the ICD-11 concept of personality functioning and severity. As the LPFS-SR is by its nature a measure of personality severity, it is an obvious candidate measure to help validate ICD-11 personality functioning. Another possible limitation is the comparison of the STIPO-R with the LPFS-SR. While the measures are correlated and produced similar findings for group comparisons, their components do not completely overlap. For instance, the Identity components across both measures correlate at *r* = 0.62. Similarly, the STIPO-R Object Relations component correlates between *r*s of 0.47–0.50 with the LPFS-SR scales, which ostensibly are aspects of object relations. The moderate degree of correlation may reflect methodological differences, as method effects are known to attenuate the degree of correlation between two similar constructs. Alternatively, these scales partially overlap in content, indicating that there are aspects of one scale that might be associated with clinically relevant outcomes that are not found in the other scale. Though it is beyond the scope of this paper, a factor analysis of this data might prove useful to determine if the shared variance among scales can be attributed to isolated factors, or whether the various scales cross-load on other factors thus demonstrating their heterogeneity.

## Conclusion

The current study sought to validate the ICD-11 conceptualization of personality functioning by evaluating the relationship of the LPFS-SR with the STIPO-R. Overall, the LPFS-SR scales were correlated with all of the STIPO-R dimensions. Additionally, both the STIPO-R and LPFS-SR scales differentiated patients with PDs, patients without PDs, and University controls, with higher levels of impairment on the LPFS-SR and STIPO-R being associated with more pervasive levels of pathology (i.e., the presence of a personality disorder). Assessing levels of personality functioning in the diagnostic manuals appears to be gaining more ecological validity, particularly as such findings appear to replicate cross-culturally.

## Data Availability Statement

The raw data supporting the conclusions of this article will be made available by the authors, without undue reservation.

## Ethics Statement

The studies involving human participants were reviewed and approved by The Research Ethics Committee of the University of Kurdistan (IR.UOK.REC.1397.014). The patients/participants provided their written informed consent to participate in this study.

## Author Contributions

AN: study conceptualization, data collection, and data preparation. AH: study conceptualization, data collection, data analysis, and report writing. SH: report writing and final proofreading. All authors contributed to the article and approved the submitted version.

## Conflict of Interest

The authors declare that the research was conducted in the absence of any commercial or financial relationships that could be construed as a potential conflict of interest.
