# Supplementary material for: The Construct Validity of the ICD-11 Severity of Personality Dysfunction Under Scrutiny of Object-Relations Theory
Source: Front Psychiatry. 2021 Jul 22;12:648427. doi: 10.3389/fpsyt.2021.648427 (PMC8340676; doi:10.3389/fpsyt.2021.648427)
Supplement: Supplementary file 1 [file Table_1.DOCX]

| **Supplementary Table 1**  *Demographic Characteristics for the College Student and Clinical Samples (N =650)* | | | |
| --- | --- | --- | --- |
| Groups | Characteristics | *n* (%) | Age range (*M, SD*) |
| College sample  (*n* = 231) | Female | 138 (59.7) | 18-58 years (28.05, 6.08) |
|  | Male | 92 (39.8) |  |
|  | Unidentified | 1 (.7) |  |
|  | Education |  |  |
|  | ≤ High School | 10 (4.3) |  |
|  | High School | 20 (8.7) |  |
|  | Undergraduate Students | 62 (26.8) |  |
|  | Masters Students | 115 (49.8) |  |
|  | Ph.D. candidates | 19 (8.2) |  |
|  | Unidentified | 5 (2.2) |  |
| Hospitalized Sample  (*n* = 419) | Female | 142 (33.9) | 18-55 years (29.56, 7.65) |
|  | Male | 276 (65.9) |  |
|  | Unidentified | 1 (.2) |  |
|  | Personality Disorders |  |  |
|  | Paranoid | 12 (2.9) |  |
|  | Schizoid | 4 (1) |  |
|  | Schizotypal | 7(1.7) |  |
|  | Antisocial | 26 (6.2) |  |
|  | Borderline | 198 (47.3) |  |
|  | Histrionic | 5 (1.2) |  |
|  | Narcissistic | 4 (1) |  |
|  | Avoidant | 4 (1) |  |
|  | Dependent | 1 (.2) |  |
|  | Obsessive-Compulsive | 6 (1.4) |  |
|  | Other Disorders |  |  |
|  | Bipolar type I | 58 (13.8) |  |
|  | Major Depression | 46 (11.0) |  |
|  | Obsessive Compulsive | 26 (6.2) |  |
|  | General Anxiety | 6 (1.4) |  |
|  | Social Anxiety | 4 (1) |  |
|  | Panic | 3 (.7) |  |
|  | Anorexia | 2 (.5) |  |
|  | Somatic Symptom | 4 (.9) |  |
|  | PTSD | 3 (.7) |  |
|  | Education |  |  |
|  | ≤ High School | 132 (31.5) |  |
|  | High School | 180 (43) |  |
|  | Undergraduate | 63 (15) |  |
|  | Master of Sciences | 9 (2.1) |  |
|  | Ph.D. | 5 (1.2) |  |
|  | Unidentified | 30 (7.2) |  |
